# Nitric oxide donors improve the ovulation and pregnancy rates in anovulatory women with polycystic ovary syndrome treated with clomiphene citrate: A RCT

**Published:** 2016-01

**Authors:** Ahmad Mahran, Ayman Abdelmeged, Hossam Shawki, Abdelrazek Moheyelden, Asmaa Mohamed Ahmed

**Affiliations:** 1 *Department of Obstetrics and Gynecology, Faculty of Medicine, Minia University,* *Minia, Egypt.*; 2 *Department of Obstetrics and Gynecology, Beni-Mazar General hospital, Minia, Egypt.*

**Keywords:** *Polycystic ovary syndrome*, *Clomiphene citrate*, *Nitric oxide donors*

## Abstract

**Background:**

Clomiphene citrate (CC) is the first line agent used for ovulation induction in patients with polycystic ovarian syndrome (PCOS). However, there is marked discrepancy between the ovulation and pregnancy rates achieved, which may be attributed to the undesirable effect of CC on cervical mucus and endometrium.

**Objective:**

The aim of this study was to evaluate the effect of Isosorbid monoitrate (ISMN) as nitric oxide (NO) donors on the ovulation and pregnancy rates in an ovulatory women with PCOS treated with CC.

**Materials and Methods:**

Ninety patients with PCOS were randomly allocated into three groups. Patients in group A) were treated with 100 mg CC for five days starting from the fifth day of the cycle. Patients in group B) and C) received 10 mg and 20 mg of ISMN respectively in addition to CC, applied vaginally till the diagnosis of ovulation.

**Results:**

There was a significant increase in the ovulation and pregnancy rates in the patients treated with CC+ISMN as compared with patients treated with CC alone (p< 0.001).

**Conclusion:**

Concomitant use of NO with CC seems to improve the ovulation and pregnancy rates in the patients with PCOS with no significant increase in side effects as compared with CC alone.

## Introduction

Polycystic ovary syndrome (PCOS) is considered as one of the most common endocrinal disorders. It affects about 6.6-8% of women in reproductive age. It is the leading cause of nearly 75% of cases of anovulatory infertility (1). Clomiphene Citrate (CC) is a selective estrogen receptor modulator. It is the first choice treatment for ovulation induction in anovulatory women with PCOS. The mechanism of action of CC is not exactly known, but may be through increasing follicle-stimulating hormone (FSH) secretion because of blocking of the negative feedback mechanism to the hypothalamus and pituitary. CC has the advantages of being used orally, being of low cost, having few adverse effects, not needing close monitoring in addition to being thoroughly studied (2). Nitric oxide (NO) is a small molecule composed of one atom of nitrogen and oxygen. It is an uncharged molecule with an unpaired electron; a characteristic makes it an ideal messenger diffuses freely across membranes (3). 

It works through an autocrine action immediately on the cell from which it is released with a biological half-life of few seconds. It is synthesised by oxidation of L-arginine by the enzyme NO-synthetase (NOS) (4). NO plays an important role in female reproduction. It is believed to be involved in follicular development as its level increases during follicle growth and decreases immediately after ovulation (5). It also regulates endometrial functions. It mediates spiral arterial changes in decidualization and promotes embryo implantation (6). The aim of this study was to evaluate the effect of treatment with NO donors on the ovulation and pregnancy rates in an ovulatory women with PCOS treated with CC.

## Materials and methods

This study is a randomized controlled trial involving ninety PCOS women among those attending fertility units in two hospitals, Minia Maternity University Hospital and Beni-Mazar General Hospital for fertility treatment. It was conducted in the period between July 2012 to January 2014. Inclusion criteria were: a) age between 20-39 years and b) diagnosis of PCOS based on the Rotterdam criteria (7), in which at least two of the following three criteria were met: 1) oligo or anovulation, 2) clinical or biochemical hyperandrogenaemia, 3) polycystic ovaries (>12 follicles <10 mm and/or ovarian volume >10 ml per ovary by vaginal ultrasound). Hyperprolactinaemia, thyroid dysfunction, Cushing’s syndrome, congenital adrenal hyperplasia, an adrenal or ovarian tumor were excluded before enrolment in the study. 

The exclusion criteria were included, a) patients with uterine pathology as fibroids, b) tubal factor of infertility diagnosed by hysteroslpingography (HSG) or laparoscopy, c) male factor infertility, and d) patients with any contraindications for CC and NO such as chronic liver and renal disease, known cardiac disease and migraine. The study was approved by the Ethical Committee of the Faculty of Medicine, Minia University, Egypt. All the patients provided written informed consent before enrolling in the study.

**Randomization**

The study was explained to all eligible patients and they were offered to take part in the study and given a patient information sheet. They were given enough time to think about. In the next visit, those who were accepted to take part in the study had given an informed consent. Patients were randomized into three groups.

Group (A): treated with CC 100 mg for five days starting from cycle day five.

Group (B): treated with CC 100 mg for five days starting from cycle day five in addition to 10 mg Isosorbid monoitrate (ISMN) tablets (Effox, Minapharm, Egypt under licence of Shwarz Pharma, Germany) applied vaginally from day 2 to day 15 of the cycle.

Group (C): treated with CC 100 mg for five days starting from fifth day of cycle in addition to 20 mg ISMN tablets applied vaginally from day 2 to day 15 of the cycle.

Randomization was done simply using sealed envelopes. The subject allocation was neither blinded to the patients nor to the physicians and investigators.

**Cycle monitoring**

**Vaginal ultrasonography**

Follicular growth in all patients was assessed using transvaginal ultrasound scans done every other day starting from cycle day 12 (Mindray 6900 with frequency 7 MHz). Presence of one or more follicle ≥18 mm that disappeared or changed in shape in subsequent scans was considered as an evidence of ovulation. Endometrial thickness was measured at the thickest point between the two basal layers on the anterior and posterior uterine walls.

**Cervical mucus scoring**

Cervical mucus was collected through Cusco speculum examination using sterile swab. It was performed on the day of folliculometry scan when a leading follicle reached 18 mm. Cervical mucus assessment was done using five parameters as shown in table I (8). The patients were advised to have intercourse every other day when a leading follicle reached 16 mm. 

Pregnancy was diagnosed by serum β-hCG performed 14 days after the expected time of ovulation. Treatment was continued for three cycles and discontinued earlier if pregnancy was achieved. 

Patients were asked to record any side effect during the treatment. Data were collected during each visit and recorded in data collection forms. Subject withdrawal: Patients were informed that they could withdraw their consent from the study at any time without giving any reason.

**Outcome measures**

The primary outcome measures of the study were:

-Ovulation rate per treatment cycle.

-Pregnancy rate per treatment cycle.

The secondary outcome measures were:

-Number of mature follicles.

-Endometrial thickness.

-Side effects recorded with the treatment

**Statistical analysis**

**Figure 1:**
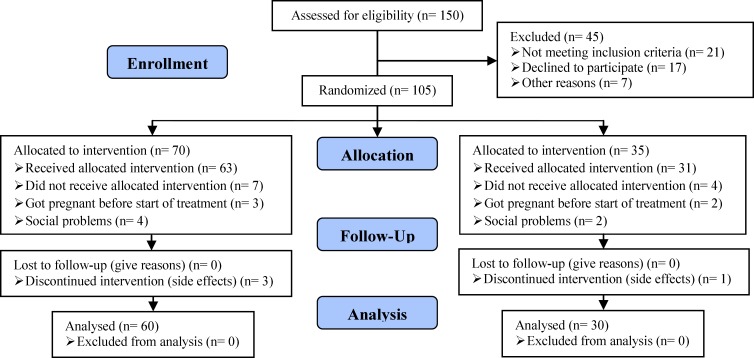
Consort flow diagram

Sample size was calculated to prevent type II error. The ovulation rate with CC in the units where the study was conducted was 40%. To be of clinical significance, we assume that the ovulation rate after the addition of ISMN needs to be 80%. Based on these date, we would need to study 27 patients in each group to be able to reject the null hypothesis that the rates for the study and control groups are equal with a probability of 80%. The type one error probability associated with this test for the null hypothesis is 0.05. To compensate for discontinuation, we recruited 50 patients in each group. The study flow chart is shown in figure 1.

Statistical analysis was performed using Statistical Package for the Social Sciences, version 17.0, SPSS Inc, Chicago, Illinois, USA (SPSS). 

Data were described in terms of mean±SD (standard deviation) for continuous variables and frequencies (number of cases) and percentages for categorical data. One way ANOVA test and Independent Student‘s *t*-test were used to compare quantitative variables, Chi square test, Fischer's exact test and Z test (test of proportions between two groups) were used to compare categorical data and Mann Whitney test was used for non-parametric data. P-value<0.05% was considered significant.

## Results

The study included 90 an ovulatory women with PCOS received 227 cycles of treatment; 81 cycles in group A, 74 cycles in group B and 72 cycles in group C. There was no significant difference in the demographic, hormonal, or sonographic features between the three groups. Characteristics of the study population are summarized in table II. There was a significant increase in the number of mature follicles, endometrial thickness and improvement of cervical mucus scores in group B (CC and 10 mg ISMN) and C (CC and 20 mg ISMN) as compared with group A (CC only). The ovulation and pregnancy rates were higher in group B and C as compared with group A. However, increase in the ISMN dose from 10 mg (group B) to 20 mg (group C) was not associated with any significant difference. Comparison between outcome measures among the three groups is shown in table III. 

Regarding the side effects recorded during the treatment, there were significantly more cases of headache, flushing and palpitation in group B and C as compared with group A. There were two cases in group C recorded syncopal attacks and that was statistically significant. 

On the other hand, there was a significant increase in ovarian cyst formation in group A compared with group B and C. Otherwise, there was no significant difference between the three groups regarding other side effects (Table IV).

**Table I:** Cervical mucus score

**Score**	**0**	**1**	**2**	**3**
Quantity	dry	0.1-0.2	0.3-0.4	>0.5
Viscosity	---	high	moderate	low
Spinnparkeit	> 3 cm	3-6 cm	7-9 cm	10-12cm
Ferning	nil	few	moderate	sufficient
Degree of external os opening	----	closed without mucous	closed with mucous	opened with mucous

Spinnparkeit: ability to pull the mucus into threads without breakage. Ferning: fern like pattern when allowing spread mucus on a dry clean slide to dry in air.

**Table II:** Characteristics of the study population

	**Group A (n= 30)**	**Group B (n= 30)**	**Group C (n= 30)**	**p-value**
Age (years)	26.1±4	27.5±4.3	26±5.4	0.37
Cycle length (days)	37.4±3.2	8.1±2.9	36.9±3.3	0.69
BMI (kg/m^2^)	27.1±4.7	26.8±4.4	26.1±4.2	0.67
Infertility type				
Primary	26 (86.7%)	28 (93.3%)	27 (90%)	0.69
Secondary	4 (13.3%)	2 (6.7%)	3 (10%)	0.38
Infertility duration (years)	4.2±2.2	4.6±2.9	4.6±3	0.85
Ovarian volume (cm^3^)	12 ± 3.7	12.4 ± 2.9	11.8 ± 3.5	0.87
AFC	28.4 ± 5.6	30.1 ± 6.1	31.1 ± 4.2	0.76
FSH (IU/l)	6.7±1.3	6.5±1.4	6.6±1.5	0.79
LH (IU/l)	11±1.5	11.8±1.4	10.9±1.6	0.82
Testosterone (ng/ml)	3.1 ± 1.1	2.9 ± 1.3	3.2 ± 1.1	0.74
SHBG (ng/ml)	36.3 ± 5.3	34.7 ± 4.9	40.1 ± 4.5	0.43
FAI	8.5 ± 1.2	8.3 ± 1.3	7.9 ± 1.3	0.54

Data are shown as mean±SD or percentage.

AFC: Antral follicle count

FSH: follicle stimulating hormone

LH: luteinizing hormone

SHBG: sex hormone binding globulin

FAI: free androgen index

NS: non-significant

**Table III:** Treatment outcome in the three groups

	**Group A** **(n= 81)**	**Group B** **(n= 74)**	**Group C** **(n= 72)**	**P1**	**P2**	**P3**
No. of mature follicles	0.82±0.4	1.19±0.3	1.29±0.4	< 0.001*	< 0.001*	0.09
Endometrial thickness(mm)	8.1±1.3	9.7±1.2	9.9±1	< 0.001*	< 0.001*	0.28
Cervical mucus score	4.2±0.9	6.12±1.3	6.3±1.2	< 0.001*	< 0.001*	0.93
Ovulation rate	26 (32.1%)	40 (54.5%)	43 (59.7%)	< 0.001*	< 0.001*	0.24
Pregnancy rate	7 (8.6%)	13 (17.6%	14 (19.4%)	0.026*	0.048*	0.39

Data are shown as mean±SD. The number in each group is the number of treatment cycles. P1: p value of the difference between group A and group B. P2: p value of the difference between group A and group C. P3: p value of the difference between group B and group C.

* Significant if p-value < 0.05

**Table IV:** Side effects recorded during treatment in the three groups

	**Group A** **(** **n** **= 30)**	**Group B** **(n =30)**	**Group C** **(n = 30)**	**P1**	**P2**	**P3**
Headache	5	15	18	< 0.001*	< 0.001*	0.78
Nausea and vomiting	3	5	5	0.34	1	1
Dizziness	7	10	12	0.56	0.01*	0.87
Syncope	0	0	2	1	0.003*	0.003*
Fatigue	9	8	7	0.93	0.87	0.84
Nervousness	1	1	1	1	1	1
Insomnia	4	4	5	1	0.76	0.76
Flushing	2	8	10	< 0.001*	< 0.001*	0.75
Palpitation	3	11	12	< 0.001*	< 0.001*	0.92
Hypotension	9	10	10	0.88	0.88	1
Hypersensitivity	0	0	0	1	1	1
OHSS	2	1	2	0.54	1	0.54
Breast discomfort	7	7	8	1	0.78	0.78
Ovarian cysts	8	4	4	0.01*	0.01*	1
Transient blurring of vision	2	2	2	1	1	1

Data are presented as number of cases. P1: p value between group A and group B. P2: p value between group A and group C. P3: p value between group B and group C.

* one way ANOVA test, p-value <0.05 considered significant.

## Discussion

PCOS is the most common cause of anovulatory infertility. Anovulation in PCOS is due to abnormal follicular development, which is more obvious in the late antral stages when arrest of follicular growth occurs, and is related to the abnormal endocrinal environment. However, the abnormalities of folliculogenesis are also evident in the very early stages, which are independent on gonadotropins (9). CC is the first line agent used for ovulation induction in PCOS patients. It achieves an ovulation rate of 75-80% (9). However, the conception rate is up to 22% per cycle in those ovulating with CC (11, 12). 

The exact explanation for this discrepancy between ovulation and pregnancy rates with CC is unknown, but several hypotheses have been suggested. CC has been shown by many studies to have an anti-estrogenic effect on the endometrial development, blastocyst implantation, on the quality of the oocytes/embryos and on the cervical mucus affecting sperm transport, and early embryonic development (13-15). In this study, the effect of adding ISMN was evaluated as NO donor applied vaginally on the outcome of ovulation induction with CC in women with PCOS. The results of the study showed that the number of mature follicles, endometrial thickness, cervical mucus score, ovulation and pregnancy rates were significantly higher in patients treated with CC and ISMN compared with the patients treated with CC alone without significant increase in side effects recorded. 

The findings of this study are supported by many previous studies emphasizing the role of NO in follicular maturation and endometrial growth. It has been postulated that the intra-follicular milieu in NO treated patients improved follicular growth, oocyte quality and maturation (16). NO was shown to be involved in follicle maturation and ovulation and to enhance vasodilatation which is responsible for follicle selection and maturation in both spontaneous and stimulated in-vitro fertilization cycles (17-19). NO is believed to support and maintain the decidualization process and plays a crucial role in implantation (20, 21). 

NO can counteract the adverse effect of CC on uterine artery blood flow and improves endometrial vascularity and receptivity leading to improvement of pregnancy rate. It was found that oral L-arginine supplementation- as a NO donor- improved ovarian response, endometrial receptivity and pregnancy rate in poor responder in-vitro fertilization patients (22, 23). The improvement in cervical mucus score with NO administration was shown by an effect that can counteract the anti-estrogenic effect of CC on cervical mucus and improves pregnancy rate (24, 25). 

There are very few studies addressing the effect of concomitant use of NO donors with CC in PCOS patients. In one study done by El-Berry and Razik, similar results were obtained with higher ovulation and pregnancy rate in the patients treated with vaginal ISMN and CC as compared with the patients treated with CC alone. 

That study included 30 PCOS patients only and recommended performing larger study to support their findings (26). On the other hand, a recent study done by Ajam *et al* including 120 PCOS patients failed to find any significant difference in ovulation and pregnancy rated between the patients treated with ISMN and CC and the patients treated with CC alone (27). However, in that study both groups of patients received exogenous gonadotropins and underwent intrauterine insemination, which might have a confounding effect on their results.

## Conclusion

In conclusion, concomitant use of NO with CC seems to improve the ovulation and pregnancy rates in patients with PCOS without significant increase in side effects as compared with CC alone. These findings need to be confirmed in the context of larger randomized controlled studies.

## Note

***Registration ID in IRCT: IRCT2016011826091N1***
